# Nurr1 (NR4A2) regulates Alzheimer’s disease‐related pathogenesis and cognitive function in the 5XFAD mouse model

**DOI:** 10.1111/acel.12866

**Published:** 2018-12-04

**Authors:** Minho Moon, Eun Sun Jung, Seong Gak Jeon, Moon‐Yong Cha, Yongwoo Jang, Woori Kim, Claudia Lopes, Inhee Mook‐Jung, Kwang‐Soo Kim

**Affiliations:** ^1^ Department of Biochemistry, College of Medicine Konyang University Daejeon Korea; ^2^ Molecular Neurobiology Laboratory, Department of Psychiatry McLean Hospital Harvard Medical School Belmont Massachusetts; ^3^ Department of Biochemistry and Biomedical Sciences, College of Medicine Seoul National University Seoul Korea; ^4^ Program in Neuroscience Harvard Medical School Belmont Massachusetts

**Keywords:** 5XFAD mouse, agonist, Alzheimer’s disease, amyloid plaques, Nurr1

## Abstract

The orphan nuclear receptor Nurr1 (also known as *NR4A2*) is critical for the development and maintenance of midbrain dopaminergic neurons, and is associated with Parkinson's disease. However, an association between Nurr1 and Alzheimer's disease (AD)‐related pathology has not previously been reported. Here, we provide evidence that Nurr1 is expressed in a neuron‐specific manner in AD‐related brain regions; specifically, it is selectively expressed in glutamatergic neurons in the subiculum and the cortex of both normal and AD brains. Based on Nurr1’s expression patterns, we investigated potential functional roles of Nurr1 in AD pathology. Nurr1 expression was examined in the hippocampus and cortex of AD mouse model and postmortem human AD subjects. In addition, we performed both gain‐of‐function and loss‐of‐function studies of Nurr1 and its pharmacological activation in 5XFAD mice. We found that knockdown of Nurr1 significantly aggravated AD pathology while its overexpression alleviated it, including effects on Aβ accumulation, neuroinflammation, and neurodegeneration. Importantly, 5XFAD mice treated with amodiaquine, a highly selective synthetic Nurr1 agonist, showed robust reduction in typical AD features including deposition of Aβ plaques, neuronal loss, microgliosis, and impairment of adult hippocampal neurogenesis, leading to significant improvement of cognitive impairment. These in vivo and in vitro findings suggest that Nurr1 critically regulates AD‐related pathophysiology and identify Nurr1 as a novel AD therapeutic target.

## 1 INTRODUCTION

Nurr1 belongs to the nuclear receptor subfamily 4A (NR4A), which is comprised of *NR4A1*, *NR4A2*, and *NR4A3* (also known as Nur77, Nurr1, and Nor1, respectively) (Pearen & Muscat, 2010). Nurr1 was identified as a member of the nuclear receptors (NRs) family in the 1990s and was shown to be robustly expressed in various regions of the central nervous system (Zetterstrom, Williams, Perlmann, & Olson, 1996). Remarkably, subsequent studies demonstrated that Nurr1 is essential for the development and maintenance of midbrain dopaminergic (mDA) neurons (Castillo et al., 1998; Kadkhodaei et al., 2009; Zetterstrom et al., 1997). Although Nurr1 and other NR4A members are classical NRs with a potential ligand‐binding domain (LBD) showing high sequence homology with those of other NRs, no endogenous/native ligands have yet been identified, and therefore, they have been designated orphan NRs (Pearen & Muscat, 2010). Despite this, our recent findings showed that small molecules (e.g., amodiaquine (AQ) and chloroquine (CQ)) can directly interact with Nurr1 and activate its transcriptional function (Kim et al., 2015), suggesting that these synthetic agonists can be used to pharmacologically activate Nurr1.

While Nurr1’s functional roles are well established in mDA neurons, given its prominent expression in other brain areas, it is reasonable to speculate that Nurr1 may play functional roles beyond those in mDA neurons. Indeed, multiple lines of recent evidence suggest that Nurr1 plays important roles in diverse brain functions, ranging from neuroprotection to cognitive functions, through many brain areas (Hawk & Abel, 2011; McNulty et al., 2012; Volakakis et al., 2010). These findings, and in particular Nurr1’s role in synaptic plasticity and learning and memory in the hippocampus, prompted us to hypothesize that Nurr1 may be involved in the pathogenesis of AD. In support of this notion, we recently reported that Nurr1 is highly co‐expressed with amyloid beta (Aβ) in 5XFAD mice, a mouse model of AD (Oakley et al., 2006), at early stages and that Nurr1‐expressing cells decline in an age‐dependent manner (Moon et al, 2015). In addition, other recent studies have reported that the expression level of Nurr1 is significantly diminished in amyloid beta (Aβ)‐treated neuronal cells (Terzioglu‐Usak, Negis, Karabulut, Zaim, & Isik, 2017), animal models (Espana et al., 2010; Parra‐Damas et al., 2014), and in postmortem brains of human AD patients (Parra‐Damas et al., 2014).

In the present study, we further examined the potential link between Nurr1’s expression and AD brain pathology in normal and in 5XFAD mice. Interestingly, we found a striking co‐expression pattern between Nurr1 and Aβ in glutamatergic neurons of the brain areas associated with AD pathogenesis, namely in the subiculum and the frontal cortex (Carlesimo et al., 2015; Hyman, Van Hoesen, Damasio, & Barnes, 1984). Nurr1 expression in glutamatergic neurons was significantly compromised in 5XFAD mice in an age‐dependent manner, supporting Nurr1’s association with AD pathogenesis. In addition, Nurr1 expression was also significantly compromised in postmortem human AD brains, compared to those of healthy subjects. In order to delineate the functional roles of Nurr1 in AD pathogenesis, we used both genetic (i.e., gene knockdown and overexpression) and pharmacological approaches (using Nurr1’s synthetic agonists) in 5XFAD mice and examined the functional effects of these manipulations.

## 2 MATERIALS AND METHODS

### 2.1 Animals and ethics statement

C57BL/6J mice, B6SJLF1/J mice, and five familial AD mutation (5XFAD) transgenic mice (Tg6799) were purchased from Jackson Laboratory (Bar Harbor, ME, USA). 5XFAD mice overexpress mutant human amyloid precursor protein (APP) with the Swedish (K670N, M671L), Florida (I716V), and London (V717I) mutations along with mutant human presenilin 1 (PS1) with two FAD mutations (M146L and L286V). These transgenes are regulated by the Thy1 promoter in neurons. All animals were handled according to the McLean's Institutional Animal Care and Use Committee and followed the National Institutes of Health guidelines.

### 2.2 Stereotactic injection

During stereotactic injection, mice were anesthetized with isoflurane using the SomnoSuite^®^ Low‐Flow Anesthesia System (Kent Scientific Corporation, Torrington, CT, USA). The virus was stereotactically introduced into the subiculum (−3.4 mm anterior–posterior, ±2.0 mm medial–lateral, and −1.75 mm dorsal–ventral relative to the bregma) of the hippocampus according to the parameters described in Paxinos and Franklin's “The Mouse Brain in Stereotaxic Coordinates” (Paxinos, 2013). The coordinates of stereotactic injection and gene delivery were validated by immunofluorescence staining with Nurr1 and GFP expression (Supporting Information Figure S1).

### 2.3 Treatment of 5XFAD mice with the synthetic Nurr1 agonist amodiaquine

We intraperitoneally treated 5XFAD mice with AQ (20 mg/kg; Sigma‐Aldrich, St. Louis, MO, USA) twice daily for 2 weeks. In prophylactic treatment, at 4 weeks after the last AQ injection, we conducted histological analyses to examine Aβ plaques deposition, neuronal loss, adult hippocampal neurogenesis, and neuroinflammation. In therapeutic treatment, behavioral analyses were conducted at 2 and 4 weeks after the last treatment with AQ, and histological experiments were performed at 4 weeks after the last AQ injection (Supporting Information Figure S2).

### 2.4 Y‐maze test

The Y‐maze task was conducted as previously described by us (Jeon et al., 2018).

### 2.5 Preparation of mouse brain tissue and immunofluorescence labeling

Mice were anesthetized, transcardially perfused with 0.05 M phosphate‐buffered saline (PBS) and then transcardially fixed with ice‐cold 4% formaldehyde (Sigma‐Aldrich) in 0.1 M phosphate buffer. The perfusion‐fixed brains were processed as previously described (Jeon et al., 2018). For double immunofluorescent labeling of Nurr1 with several histological markers, such as EAAC1 (excitatory amino acid carrier 1), ChAT (choline acetyltransferase), α‐GABA (gamma‐aminobutyric acid A Receptor alpha 1), 4G8, NeuN (neuronal nuclear antigen), GFAP (glial fibrillary acidic protein), or Iba‐1 (ionized calcium‐binding adapter molecule 1), the coronal brain sections containing the subiculum or deep cortical layer were taken from each brain. For 4G8 staining, the tissues were pretreated with 70% formic acid for 20 min. Sections were rinsed three times for 10 min with PBS. The brain slices were then double‐stained with a rabbit anti‐Nurr1 antibody developed by us (Leblanc et al., 2015) and with the off‐the‐shelf antibodies, including mouse anti‐4G8 antibody (1:1,000; BioLegend, San Diego, CA), mouse anti‐EAAC1 antibody (1:200; Abcam, Cambridge, UK), goat ChAT antibody (1:200; EMD Millipore, Burlington, MA, USA), mouse α‐GABA antibody (1:1,000, 1:200; EMD Millipore), mouse anti‐NeuN antibody (1:200; Merck Millipore, Billerica, MA, USA), rat anti‐GFAP (1:100; Thermo Fisher Scientific, Waltham, MA USA), and mouse anti‐Iba‐1 antibody (1:500; EMD Millipore) overnight at room temperature in PBS with 0.3% Triton X‐100 (Sigma‐Aldrich) and 1% bovine serum albumin (Sigma‐Aldrich). After washing three times in PBS, the sections were incubated with an Alexa 594‐conjugated donkey anti‐rabbit IgG (1:200; Molecular Probes, Eugene, OR, USA), Alexa 488‐conjugated donkey anti‐mouse IgG (1:200; Molecular Probes), Alexa 488‐conjugated donkey anti‐goat IgG (1:200; Molecular Probes), and Alexa 488‐conjugated donkey anti‐rat IgG (1:200; Molecular Probes) for 1.5 hr at room temperature with PBS containing 0.3% Triton X‐100 and DAPI (1:1,000). Immunolabeled sections were mounted on Probe‐On™ Plus Microscope Slides (Thermo Fisher Scientific, Portsmouth, NH, USA) and coverslipped using Fluoroshield™ (Sigma‐Aldrich).

### 2.6 Western blotting analyses

Fresh frozen postmortem brain samples from AD patients (Braak VI; *n* = 5) and from age‐ and sex‐matched normal subjects (*n* = 4) were provided by the Harvard Brain Tissue Resource Center. Superior frontal cortex, hippocampal formation, and substantia nigra frozen sample dissections (approximately 100 mg) from AD patients and normal controls were processed in homogenization buffer (50 mM Tris‐HCl (pH 8.0), 150 mM NaCl, 0.5 mM EDTA, 1% Triton X‐100 (Sigma, St. Louis, MO)) containing 1 mM PMSF, protease inhibitor cocktail, and phosphatase inhibitor cocktail (Thermo Fisher Scientific). Lysates were sonicated and centrifuged at 16,000 *g* for 30 min at 4°C, and the supernatants were collected and stored at −80°C before use. Equal amounts of protein sample (20 µg) were separated by PAGE and transferred to PVDF membranes. After blocking, membranes were incubated with the following primary antibodies: rabbit anti‐Nurr1 (1:1,000) and rabbit anti‐β‐actin (Abcam, Cambridge, MA; 1:5,000). Quantification of immunoreactive bands is reported as a ratio against β‐actin using ImageJ software (NIH, Bethesda, MD).

### 2.7 Statistical analysis

All data are shown as the mean ± standard error of the mean (*SEM*). GraphPad Prism 5 (GraphPad Software, CA, USA) was used for statistical significance tests. The independent *t* test was used for comparison between the two groups, and the one‐way ANOVA with post hoc Fisher's LSD test was used for comparisons with two groups or more. *p* < 0.05 was considered statistically significant.

## 3 RESULTS

### 3.1 Nurr1 is primarily expressed in glutamatergic neuron, but not in glial cells, in AD‐associated brain areas

**Figure 1:**
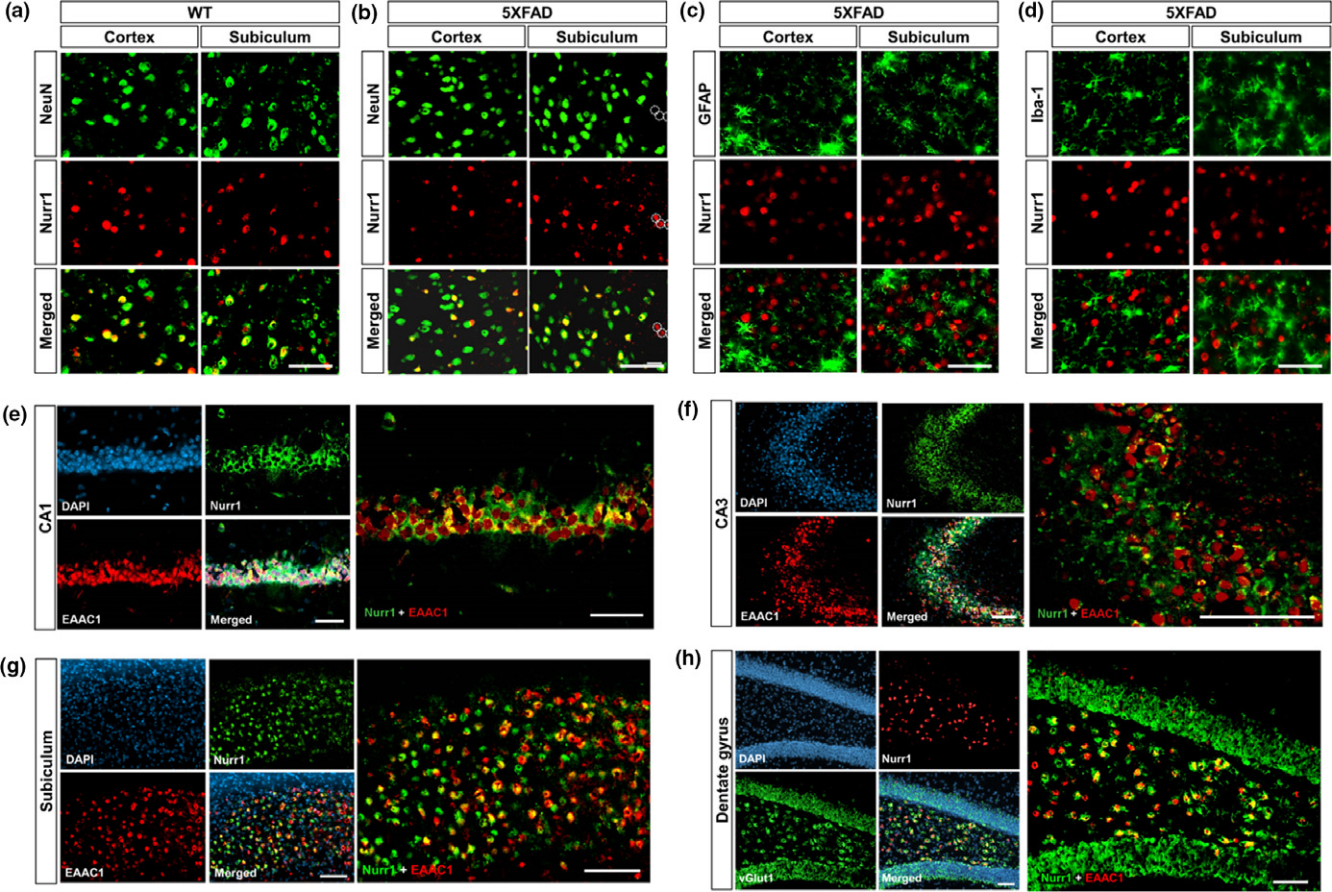
Nurr1 is cell type‐specific expressed in glutamatergic neurons in the brains of wild‐type littermates and 5XFAD mice. (a, b) Double labeling of WT and 5XFAD mice brains (4 months of age (*n* = 4–5)) with NeuN‐ and Nurr1‐specific antibodies. White circles in B indicate NeuN‐negative and Nurr1‐positive glial cells. Scale bar = 20 μm. (c) Increase in astrogliosis in the subiculum of 5XFAD as shown by staining for GFAP, an astrocyte marker. Double labeling with Nurr1 and with astrocyte marker GFAP antibodies (d) or with microglia marker Iba‐1 antibody (e) in the subiculum of 5XFAD mice. Immunostaining with antibodies against Nurr1 and EAAC1 (or vGlut1), a marker for glutamatergic neurons, in the hippocampus: e, CA1; f, CA3; g, subiculum; h, dentate gyrus; b, subiculum; c, CA1; d, CA3; e, dentate gyrus (DG). DAPI was used to stain the nuclei. Scale bar = 50 μm (a–e and g), Scale bar = 100 μm (f and h)

Although Nurr1 is known to be widely expressed in the brain (Saucedo‐Cardenas & Conneely, 1996; Zetterstrom et al., 1996), it is poorly understood what types of neuronal and/or glial cells express it other than mDA neurons (Castillo et al., 1998; Saucedo‐Cardenas et al., 1998; Zetterstrom et al., 1997). Thus, we examined Nurr1 expression in neuronal and/or glial cell types by double staining with a neuronal marker (NeuN), an astrocyte marker (GFAP), or a microglial marker (Iba‐1). We primarily analyzed the subiculum and cerebral cortex because these brain regions are prominently linked to AD with robust Aβ deposition in the brains with AD (Carlesimo et al., 2015; Hyman et al., 1984). Remarkably, we found that a great majority of Nurr1‐expressing cells co‐express NeuN in the wild‐type (WT) mice (Figure 1a). Quantification of these cell populations revealed that Nurr1‐positive and NeuN‐negative cells (which are presumably Nurr1‐expressing glia) represent only 1.0% and 3.5% of the total Nurr1‐positive cells in the subiculum and the deep cortical layers, respectively (Supporting Information Figure S4). This demonstrates that Nurr1 is mainly expressed in neuronal cells, not in resting glial cells, under non‐inflammatory conditions. We next stained the brains of 5XFAD mice with antibodies against NeuN and Nurr1 (Figure 1b and Supporting Information Figure S5). By quantifying the NeuN‐negative and Nurr1‐positive cells, we observed that Nurr1‐expressing glial cells (dotted white circles in Figure 1b) constitute 4.1% and 6.5% of Nurr1‐positive cells, respectively, in the subiculum and the deep cortical layers (Supporting Information Figure S4). These data show that the number of Nurr1‐expressing glial cells increased in both the subiculum and the deep cortical layers of 5XFAD mice, compared to those in WT littermates (Supporting Information Figure S4). However, the majority of Nurr1‐expressing cells were still neuronal in both the subiculum (95.9%) and the cortex (93.5%) of the 5XFAD mice brain. To further confirm the expression pattern of Nurr1 in the brains affected by neuroinflammation, we performed immunohistochemical co‐staining of GFAP or Iba‐1 with Nurr1 in the subiculum and the cerebral cortex of 5XFAD mice (Figure 1c,d), and again confirmed that Nurr1 is predominantly expressed in neurons in 5XFAD mice.

We next investigated what type(s) of neuronal cells express Nurr1. In particular, given the important roles of hippocampal or cerebral cortical glutamatergic neurons in AD pathogenesis (Butterfield & Pocernich, 2003; Leshchyns'ka et al., 2015; Rodriguez‐Perdigon et al., 2016), we examined co‐expression of Nurr1 and a marker for glutamatergic neurons (e.g., EAAC1 or vGlut1). Histological analysis showed that there are multiple glutamatergic Nurr1‐expressing cells in the hippocampal formation (Figure 1e–h). In addition, glutamatergic neurons expressing Nurr1 were abundant in the dentate gyrus (DG), CA1 and CA3 regions of the hippocampus (Figure 1e–h). We also confirmed Nurr1 expression in extra‐hippocampal areas, such as deep cortical layers (Supporting Information Figure S6). To further analyze cell type‐specific expression of Nurr1, we co‐stained brain tissues with antibodies against Nurr1 and GABAergic or cholinergic neuronal markers and found that the immunoreactivity between Nurr1 and α‐GABA or ChAT did not overlap at all in the subiculum (Supporting Information Figure S7).

### 3.2 Age‐dependent degeneration of Nurr1‐expressing Aβ‐positive cells in 5XFAD mice and reduced expression of Nurr1 in the hippocampus and the frontal cortex, but not in the substantia nigra, of postmortem brains of AD patients

**Figure 2:**
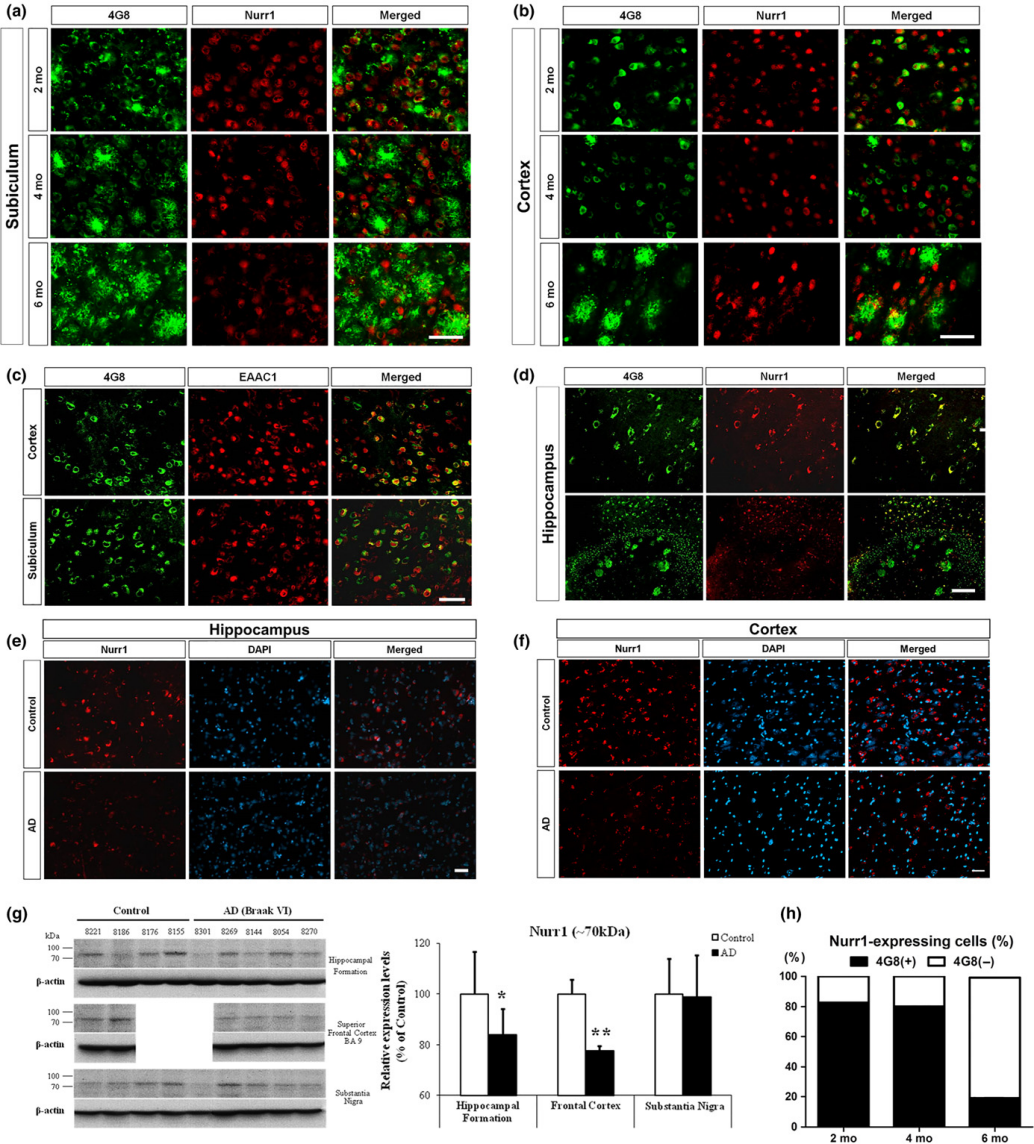
Nurr1 expression in Aβ accumulating cells in 5XFAD mouse brains and in the postmortem brains of human AD patients. Nurr1 and 4G8 double labeling in the subiculum (a) and in the cerebral cortex (b) of 2‐, 4‐ and 6‐month‐old 5XFAD mice (*n* = 4–5). (c) Immunostaining with antibodies against Nurr1 and EAAC1 in the frontal cortex and in the subiculum of 5XFAD mice at 2 months of age. (d) Nurr1 and 4G8 double labeling of the hippocampus of postmortem human AD brains. Analysis of Nurr1‐positive cells in the hippocampus (e) and frontal cortex (f) of control and AD human brains. All brain tissues were counterstained with DAPI. (g) Western blot analysis and quantification of Nurr1 expression in the hippocampal formation, superior frontal cortex, and substantia nigra of normal and AD patient brains. (h) Classification of Nurr1‐expressing cells according to Aβ expression in the subiculum of 5XFAD mice. *p < 0.05 and **p < 0.01 versus healthy control brains. Scale bar = 50 μm

We next investigated how Nurr1‐expressing neurons change during disease progression in 5XFAD mice. Although we previously showed that Nurr1‐expressing cells decreased in aged 5XFAD mice (Moon et al, 2015), it is unknown whether Nurr1 expression is altered in glutamatergic neurons in the brains of AD animal models. We first examined expression patterns of Nurr1 and Aβ and found that they are highly co‐localized in the subiculum and the frontal cortex of 5XFAD mice (Figure 2a,b, and Supporting Information Figure S8–S10). In addition, a great majority of 4G8‐positive cells co‐expressed EAAC1 (Figure 2c). Our histological analysis showed that a majority of Nurr1‐expressing cells in the subiculum of 5XFAD mice express Aβ in the early stages of Aβ deposition: The percentage of Aβ (4G8)^+^ cells among Nurr1‐expressing cells was 82.7% and 80.2%, respectively, in 2‐ and 4‐month‐old animals (Figure 2h). However, at a later stage (6 months old) with massive accumulation of Aβ, the proportion of Nurr1‐positive cells expressing Aβ dramatically decreased to 19.1% in the subiculum of 5XFAD mice. These data suggest that Nurr1‐expressing Aβ‐positive glutamatergic neurons progressively degenerate in the hippocampal formation of 5XFAD mice.

We next investigated expression patterns of Nurr1 in the postmortem brains of AD and healthy subjects by double staining of 4G8 with Nurr1. As shown in Figure 2d, we found that 4G8‐positive cells are highly co‐localized with Nurr1 expression in the hippocampus, which is in agreement with our results in 5XFAD mice. Moreover, the number of Nurr1‐containing cells was reduced in the hippocampus and the frontal cortex compared to healthy control brains (Figure 2e,f). Consistent with these findings, western blot analyses showed that Nurr1 expression was significantly reduced in the hippocampus and in the superior frontal cortex of AD brains compared to healthy brains (Figure 2g). Notably, however, our analysis showed that Nurr1 expression is not altered in the substantia nigra of AD brains. Thus, our results show for the first time that Nurr1 expression is significantly reduced in a pathophysiologically relevant pattern in both animal models of AD and AD patients, further supporting a potential functional role(s) of Nurr1 in AD pathogenesis.

### 3.3 Effects of Nurr1 knockdown and overexpression on histopathological manifestations of AD in 5XFAD mice

**Figure 3:**
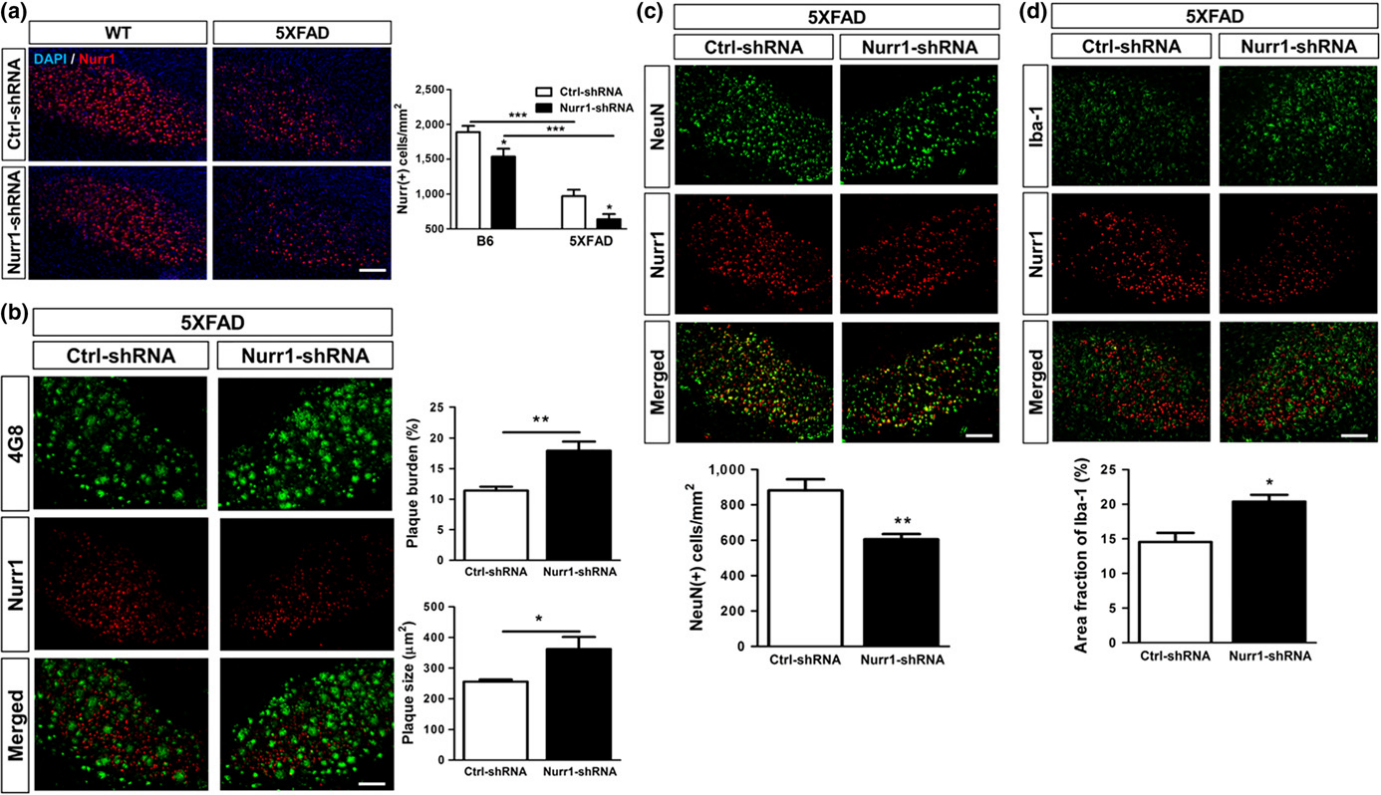
Functional effects of Nurr1 knockdown on histopathological manifestations of AD in 5XFAD mice. (a) Injection of sh‐Nurr1 lentivirus significantly reduces expression of Nurr1 in the subiculum of both WT and 5XFAD mice (*n* = 3). ***p < 0.001, WT versus 5XFAD. ^#^ *p* < 0.05, Ctrl‐shRNA versus Nurr1‐shRNA. (b) Nurr1 knockdown by Nurr1‐shRNA lentivirus significantly increases the burden and size of Aβ‐plaques in the subiculum of 5XFAD mice. (c) Nurr1 knockdown accelerates reduction of NeuN (+) cells in the subiculum of 5XFAD mice. (d) Nurr1 knockdown increased the number of Iba‐1 (+) cells. *p < 0.05, **p < 0.01, ***p < 0.001, two‐paired *t* test comparing Ctrl‐shRNA and Nurr1‐shRNA in 5XFAD mice. Scale bar = 100 μm

**Figure 4:**
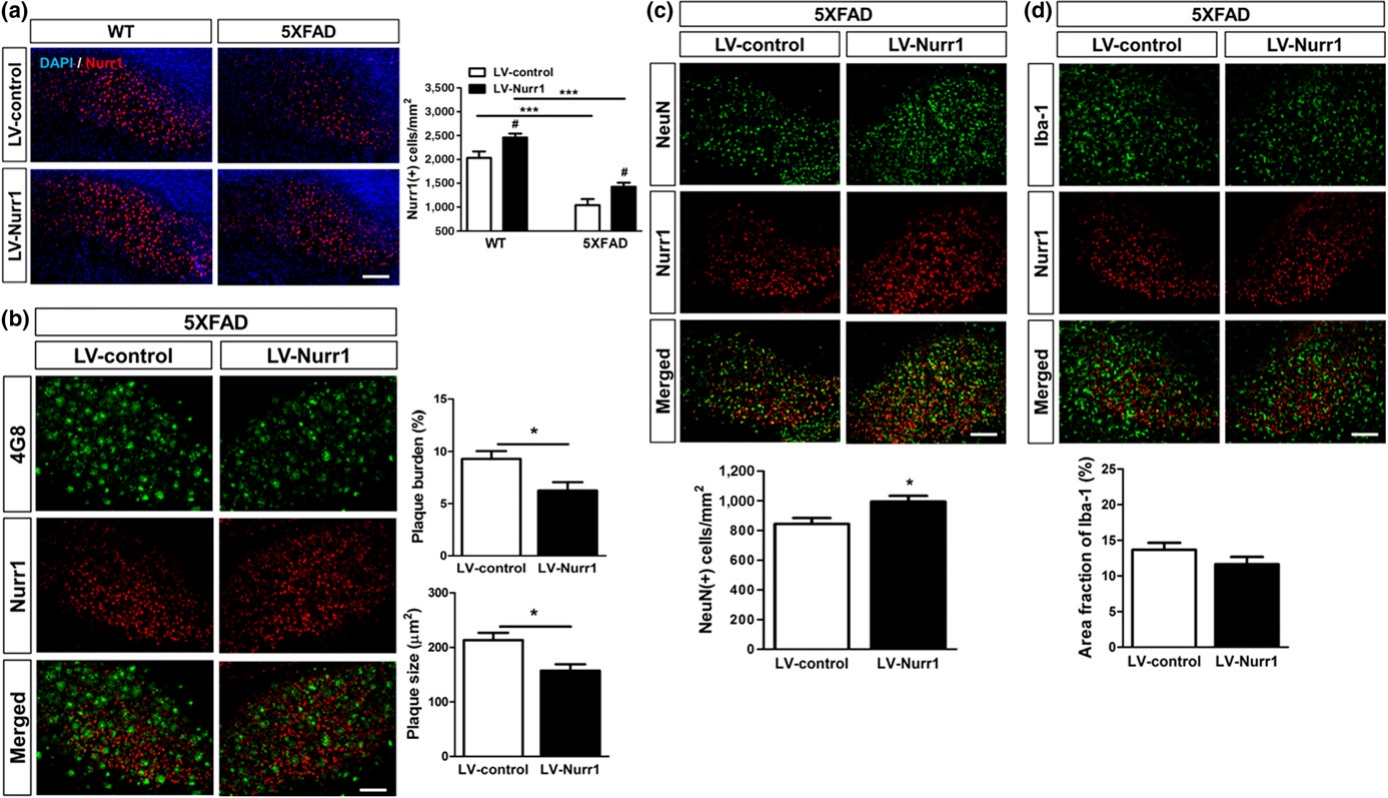
Functional effects of Nurr1 overexpression on AD‐related pathology in 5XFAD mice. (a) Injection of lentiviral vectors expressing Nurr1 significantly increases expression of Nurr1 in the subiculum of both WT and 5XFAD mice (*n* = 3). ***p < 0.001, WT versus 5XFAD. ^#^ *p < *0.05, LV‐control versus LV‐Nurr1. (b) Nurr1 upregulation significantly decreases the burden and size of Aβ‐plaques in the subiculum of 5XFAD mice. (c) Nurr1 overexpression inhibits the reduction of the number of NeuN (+) cells in the subiculum of 5XFAD mice. (d) Nurr1 overexpression decreases microgliosis. *p < 0.05, two‐paired *t* test comparing LV‐control and LV‐Nurr1 in 5XFAD mice. Scale bar = 100 μm

We next explored whether this association between Nurr1 expression and the progression of AD pathology reflects a mechanistic involvement of Nurr1 in the pathogenesis of the disease by examining the effects of either downregulation and/or overexpression of Nurr1. First, we stereotactically injected Nurr1‐shRNA into the subiculum of 5XFAD mice. Two microliters of titrated Nurr1 shRNA lentivirus (Nurr1‐shRNA; approximately 2 × 10^9^ Tu/ml) or of scrambled shRNA lentivirus (Ctrl‐shRNA; approximately 2 × 10^9^ Tu/ml) was infused into the dorsal subiculum of WT and 5XFAD mice at 3 months of age. At 2 months postinfection, Nurr1 expression was significantly reduced in the Nurr1‐shRNA injected subiculum, compared to the Ctrl‐shRNA injected subiculum, in the WT mice (Figure 3a). The number of Nurr1‐expressing cells was much lower in the subiculum of 5XFAD than those in the normal mice, and this number was reduced even further by the Nurr1 knockdown (Figure 3a). In the subiculum with Nurr1‐shRNA injection, we found that the burden and size of the Aβ plaque were significantly increased compared to those of the Ctrl‐shRNA injected subiculum (Figure 3b). In addition, the number of NeuN‐positive cells in the Nurr1‐shRNA injected subiculum was significantly decreased compared to that in the Ctrl‐shRNA injected subiculum (Figure 3c). In contrast, the fraction of Iba‐1‐positive cells significantly increased in the Nurr1‐shRNA injected subiculum (Figure 3d). Together, these results indicate that Nurr1 knockdown resulted in acceleration of AD‐related pathology, such as Aβ deposition, neuronal loss, and microglial activation. Next, we tested the effects of Nurr1 overexpression by injecting a lentiviral vector expressing Nurr1 into the subiculum of the WT and 5XFAD mice. Two microliters of titrated Nurr1‐overexpressing lentivirus (LV‐Nurr1; approximately 2 × 10^9^ Tu/ml) or empty vector lentivirus (LV‐control; approximately 2 × 10^9^ Tu/ml) was infused into the dorsal subiculum of WT and 5XFAD mice at 3 months of age. At 2 months postinfection, we confirmed a significant upregulation of Nurr1 expression in the LV‐Nurr1 injected subiculum of both WT and 5XFAD mice compared to the LV‐control injected subiculum (Figure 4a). In the LV‐Nurr1 injected subiculum with upregulated expression of Nurr1, the burden and size of Aβ‐plaque were significantly diminished compared to the LV‐control injected subiculum in the same mouse brain (Figure 4b), while the number of NeuN‐positive cells was significantly increased (Figure 4c). The fraction of Iba‐1‐positive cells did not show a significant difference (Figure 4d).

### 3.4 Pharmacological treatment with a synthetic agonist of Nurr1, AQ, significantly ameliorates ad‐related neuropathology and improves memory impairments in 5XFAD mice

**Figure 5:**
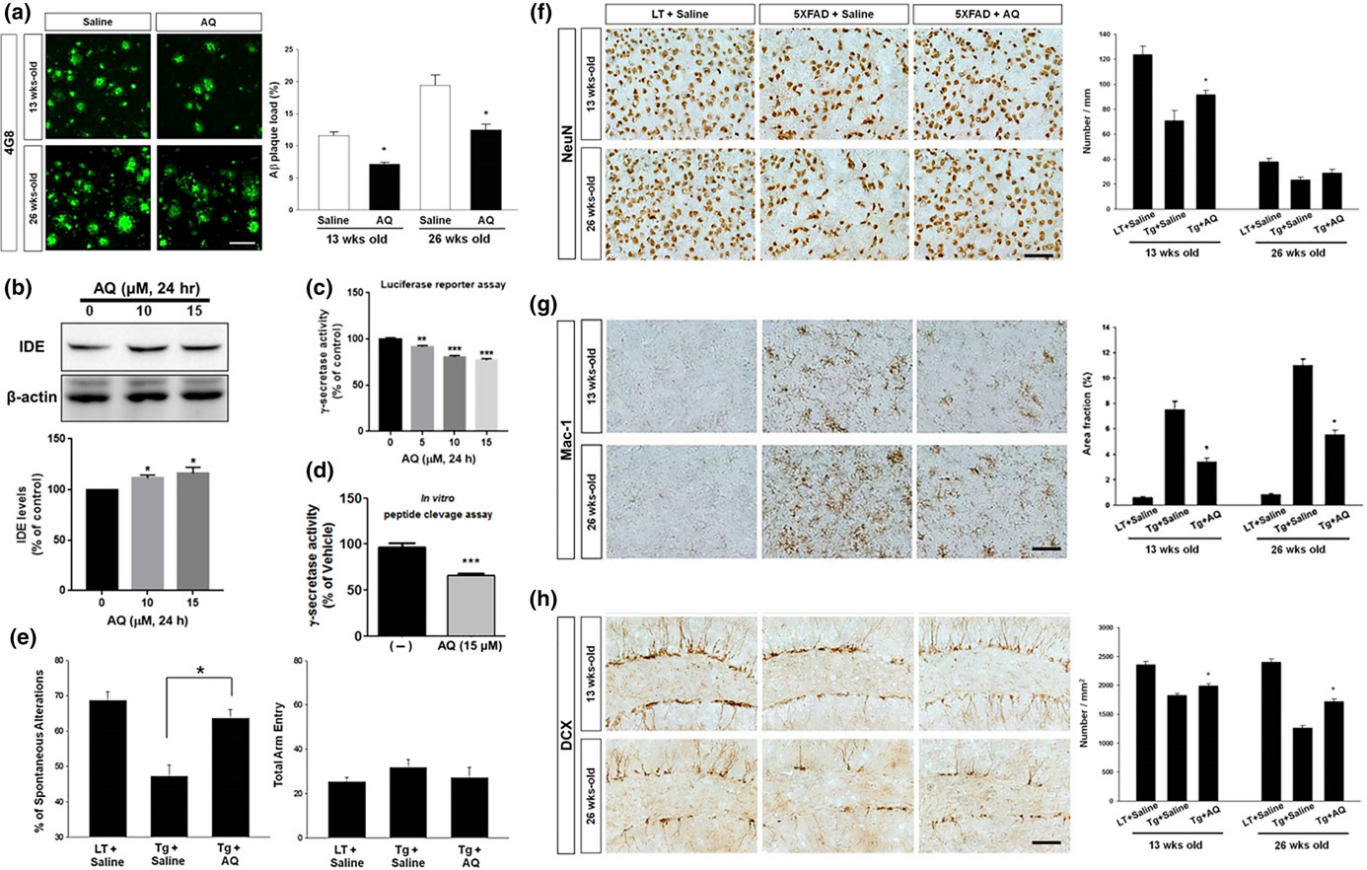
Treatment with AQ inhibits Aβ‐mediated pathology and improves cognitive function in 5XFAD mice. (a) Nurr1 agonist significantly reduced 4G8‐positive plaques in the subiculum of 5XFAD mice (*n* = 10). Scale bar = 50 μm. (b) Representative immunoblot image and quantification of IDE expression levels from AQ‐treated SH‐SY5Y cells. *p < 0.05. Cells were treated with the Nurr1 agonist, AQ, for 24 hr at the indicated concentration, and then γ‐secretase activity was measured. (c) SH‐SY5Y‐C99 cells were used for luciferase reporter assay to measure γ‐secretase activity. **p < 0.01, ***p < 0.001. (d) SH‐SY5Y cells were used for in vitro peptide cleavage assay to measure γ‐secretase activity. ***p < 0.001. (e) AQ treatment significantly improved cognitive impairments in 5XFAD mice (*n* = 10) 4 weeks after the last injection. Administration of AQ did not cause changes in general behavior or spontaneous locomotor activity. (f) AQ treatment reduced neuronal loss in the subiculum of 5XFAD mice. (g) AQ treatment reduced microgliosis in the subiculum of 5XFAD mice. (h) The effect of AQ treatment on hippocampal neurogenesis in 5XFAD mice. AQ increased the number of DCX‐positive cells in the dentate gyrus of 5XFAD mice. *p < 0.05 versus saline‐treated 5XFAD mice compared by ANOVA and post hoc Fisher's LSD test. Scale bar = 50 μm

Our loss‐of‐function and gain‐of‐function studies prompted us to hypothesize that pharmacological activation of Nurr1 may improve pathogenic features of AD. To address this hypothesis, we treated 5XFAD mice with AQ, which has been found to activate Nurr1 transcriptional function by directly interacting with its LBD (Kim, Leblanc, & Kim, 2016). First, we estimated the area occupied by Aβ, as examined by immunoreactivities against 4G8 in the subiculum of 5XFAD mice that were treated with either saline or AQ (Figure 5a). Remarkably, we found that the plaque load was significantly reduced by AQ treatment in both 13‐ and 26‐week‐old 5XFAD mice. These data indicate that AQ may have ability to reduce the accumulation of Aβ peptide in these Aβ‐overexpressing animals. To elucidate potential mechanisms, we next tested whether AQ treatment affects Aβ biosynthesis and/or clearance in vitro using the SH‐SY5Y human neuroblastoma cell line which expresses Nurr1 (Pan et al., 2008). Using western blot analysis, we found that treatment with 10 and 15 µM AQ significantly increased an Aβ‐degrading protease, insulin‐degrading enzyme (IDE) (Figure 5b). In addition, using luciferase reporter assays, we observed that AQ treatment significantly inhibited γ‐secretase activity in a dose‐dependent manner (Figure 5v). Furthermore, our in vitro peptide cleavage assay showed that AQ treatment (15 µM) significantly inhibited γ‐secretase activity in SH‐SY5Y cells (Figure 5d). These results suggest that Nurr1 activation may reduce accumulation of amyloid plaques, at least in part, by inhibition of γ‐secretase activity and enhanced degradation of Aβ via upregulation of IDE.

We next tested whether a systemic injection of AQ could improve cognitive functions in 5XFAD mice. Since 5XFAD mice exhibit subiculum‐specific neurodegeneration (Oakley et al., 2006) and the subiculum is involved in spatial working memory (Riegert et al., 2004), we measured cognitive function quantitatively using a Y‐maze task, which is widely accepted as a behavioral paradigm for evaluating spatial working memory. AQ treatment increased the levels of spontaneous alternations in 5XFAD mice 2 weeks after the last injection of AQ, but the difference was not statistically significant (Supporting Information Figure S12). Interestingly, 4 weeks after the last injection of AQ, the lowered levels of spontaneous alternations in 5XFAD mice were significantly reversed by AQ (Figure 5e). The total number of arm entries did not significantly differ in any of the groups, indicating that levels of general motor and exploratory activity in the Y‐maze were not changed. Taken together, our data show that systemic administration of AQ can rescue memory deficits in 5XFAD mice but requires a sufficient time to acquire its full effect.

To further understand the functional effects of AQ treatment, we also investigated whether it has neuroprotective effects in 5XFAD mice. A significant neuronal loss was detected in the subiculum area of both 13‐ and 26‐week‐old 5XFAD mice injected with saline, as examined by quantification of the number of NeuN‐positive cells (Figure 5f). When these mice were analyzed following AQ treatment, neuronal loss was significantly ameliorated in the subiculum of 5XFAD mice, compared to the saline‐administered 5XFAD mice (Figure 5f), suggesting that pharmacological activation of Nurr1 exerts a neuroprotective effect in an animal model of AD. Because Nurr1 is known to regulate neuroinflammation (Saijo et al., 2009), we next tested whether AQ treatment modulates microglial activation in the subiculum of 5XFAD mice, using immunostaining with antibody against Mac‐1, which is a marker for microglia. We found that the size of Mac‐1‐stained areas was robustly increased in the subiculum of 5XFAD mice compared to that of WT littermates and that there was a progressive increase in this microgliosis with age (Figure 5g). Interestingly, AQ treatment significantly diminished microglial activation in both 13‐week‐old and 26‐week‐old 5XFAD mice. In addition, we examined changes in Mac‐1 immunoreactivity in the hippocampal formation, including DG, hippocampus proper, and the subiculum, and confirmed that AQ treatment prominently ameliorated microglial activation in these areas as well (Supporting Information Figure S11). Finally, since impaired neurogenesis is another cardinal pathological feature in AD, we examined whether Nurr1 activation by AQ can enhance adult hippocampal neurogenesis in 5XFAD mice using immunostaining with an antibody against DCX, a marker for adult neurogenesis (Couillard‐Despres et al., 2005). 5XFAD mice showed a significant reduction in the number of DCX‐expressing neuroblasts in the subgranular zone at both 13 and 26 months old, compared to WT mice (Figure 5h). We found that AQ treatment resulted in a significant increase in the number of DCX‐positive neuroblasts in both 13‐week‐old and 26‐week‐old 5XFAD mice (Figure 5h). Taken together, our data suggest that AQ treatment ameliorated multiple aspects of neuropathology, correlating with cognitive improvement in 5XFAD mice.

## 4 DISCUSSION

In AD, neuronal degeneration spreads in a stereotypical fashion, with certain regions of the brain such as the subiculum exhibiting earlier degeneration, while other regions such as the cerebellum are spared until the late stages of the disease (Carlesimo et al., 2015; Haass & Selkoe, 2007; Hyman et al., 1984). This selective neurodegeneration and focal pattern of disease propagation might result, at least in part, from selective accumulation of Aβ within neurons (Braak & Del Tredici, 2016; Haass & Selkoe, 2007). Our previous findings that Nurr1 is highly expressed in the subiculum and the frontal cortex in 5XFAD mice showing the highest levels of amyloid deposition (Moon et al., 2015) suggested a functional link between Nurr1 and Aβ‐mediated pathology of AD, but did not clarify the nature of the mechanistic link. To better understand this, we first investigated what type of cells expresses Nurr1 in these Aβ accumulating regions and found that Nurr1 is highly expressed in neurons, but not in glial cells, in both normal and 5XFAD mice. Remarkably, we found that Nurr1 was specifically expressed in glutamatergic neurons of the hippocampus of healthy brains and that these Nurr1‐expressing, Aβ‐positive glutamatergic neurons degenerated in an age‐dependent manner in 5XFAD mice. Given that glutamatergic neurons in the hippocampus and the cerebral cortex are closely associated with AD pathogenesis (Butterfield & Pocernich, 2003; Francis, 2003; Revett, Baker, Jhamandas, & Kar, 2013), our findings suggest that Nurr1 plays important roles in AD pathogenesis. In support of this possibility, we found that Nurr1 knockdown resulted in a significant acceleration of AD‐related pathology, while its overexpression alleviated all of these histopathological symptoms of AD, suggesting that upregulation of Nurr1 can ameliorate AD‐related neuropathology (Supporting Information Figure S13).

Although Nurr1 is generally known to be a ligand‐independent transcription factor (Wang et al., 2003), we recently identified three FDA‐approved drugs (i.e., AQ, CQ, and glafenine) that prominently modulate Nurr1’s transcriptional function via direct interaction with its LBD (Kim et al., 2015). Among these three compounds, AQ was most potent for activating Nurr1’s transcriptional function and was highly selective. For instance, AQ did not activate the transcriptional function of other NRs tested (e.g., glucocorticoid receptor, retinoid X receptor‐α (RXRα), liver X receptor‐α, or peroxisome proliferator‐activated receptor‐α and ‐β). Thus, we used AQ as a selective pharmacological tool to examine whether Nurr1 activation can ameliorate AD‐related pathology. Remarkably, we found that administering AQ to 5XFAD mice resulted in reduced deposition of Aβ plaques in the subiculum and significantly ameliorated AD‐like pathology in 5XFAD mice (e.g., neuronal loss, microglial activation, and impairment of adult hippocampal neurogenesis), leading to significant improvement of memory deficits. Notably, recent studies showed that activation of diverse NRs (e.g., retinoid X receptor; RXR) regulate AD pathogenesis and ameliorate cognitive dysfunction in mouse models of AD (Cramer et al., 2012; Fitz, Cronican, Lefterov, & Koldamova, 2013). Since RXR is well known to heterodimerize with Nurr1 (Perlmann & Jansson, 1995), it will be of great interest whether agonists of Nurr1 and RXR may co‐operatively or synergistically influence the AD pathogenesis.

Taken together, our data suggest that reduction in Nurr1‐expressing glutamatergic neurons in the hippocampal formation may be associated with AD, and that Nurr1 activation could be a promising therapeutic strategy to treat AD.

## CONFLICT OF INTERESTS

The authors declare that they have no conflict of interest.

## AUTHORS’ CONTRIBUTION

M.M. performed and analyzed all histological data about human brain and pharmacological stimulation for Nurr1 and also involved in the design of the whole experiment and writing of the manuscripts. E.S.J. performed and analyzed all enzymatic activity about effects of pharmacologic stimulation of Nurr1 on enzymes involved in Aβ processing in vitro. S.G.J. and C.L. performed a stereotaxic injection to regulation of the expression of Nurr1 through the virus, and performed and analyzed all histological results that derived from knockdown and overexpression of Nurr1. M.C. performed and analyzed all behavioral data about pharmacological stimulation of Nurr1. Y.J. designed and produced a virus for overexpression and knockdown of Nurr1. W.K. performed and analyzed all biochemical data about Nurr1 expression in human brain. K.K. and I.M. supervised the project, developed the theory, and wrote the manuscript.

## Data Availability

Authors declare that the author provides the data to requester when there is a reasonable request for all data supporting the findings.
