# Supplementary material for: Nurr1 (NR4A2) regulates Alzheimer’s disease‐related pathogenesis and cognitive function in the 5XFAD mouse model
Source: Aging Cell. 2018 Dec 4;18(1):e12866. doi: 10.1111/acel.12866 (PMC6351845; doi:10.1111/acel.12866)
Supplement: Supplementary file 1 [file ACEL-18-e12866-s001.docx]

**Supporting Information**

**Generation of lentivirus containing scrambled or Nurr1 shRNA**

To reduce Nurr1 expression, scrambled shRNA (#RHS4346) and specific Nurr1 shRNAs (#V2LMM 43146, TAACCATCCCAACAGCTAG and #V2LMM 44007, TAAAGGAGAAGAGTGACAG) were purchased from GE Dharmacon (Lafayette, CO, USA), and pLenti mock and pLenti-mNurr1 vectors were purchased from OriGene (Rockville, MD, USA). For producing lentiviruses, shRNA plasmids (scrambled or Nurr1) or pLenti plasmids (mock or mNurr1) combined with two helper plasmids (psPAX2 and pMD2.G) were transiently transfected into HEK293T cells using PolyJet^TM^ Reagent (Rockville, MD, USA). 48 hours after transfection, the virus-containing medium was collected and centrifuged at 1000 rpm for 10 min, and subsequently filtered through a 0.45 μm filter (Millipore). To concentrate the lentivirus, the supernatant was mixed with Lenti-X concentrator (Clontech Laboratories, Mountain View, CA, USA) and the mixture was incubated at 4 °C overnight, centrifuged (3000 rpm, 45 min) at 4 °C, and suspended in PBS. Finally, the viral titer was determined using the QuickTiter™ Lentivirus Titer Kit (Cell Biolabs, San Diego, CA, USA) according to the manufacturer’s instructions.

**Human subjects and tissue processing**

Postmortem tissue blocks, containing the hippocampal formation, superior frontal cortex, and substantia nigra, from AD (Braak V-VI) patients and healthy control donors were obtained from the Harvard Brain Tissue Resource Center, Belmont, Massachusetts (Fig. S3). Human brain tissues immunohistochemistry was performed as described previously (Pantazopoulos *et al*, 2013). 40 µm-serial sections from postmortem tissue blocks were obtained using a freezing sliding microtome and stored in cryoprotectant at -20℃. For immunohistochemistry, free-floating sections were incubated in 70% formic acid for 20 min, incubated in antibodies against Nurr1 (1:500; rabbit) and 4G8 (1:1,000; BioLegend, San Diego, CA) for 48 hours, and then incubated in Alexa 594 chicken anti-Rabbit (1:500; Invitrogen, Carlsbad, CA) and Alexa 488 goat anti-mouse (1:500; Invitrogen, Carlsbad, CA) for 2 hours at room temperature. Subsequently, the brains were incubated in DAPI solution for 2 hours at room temperature to visualize nucleic acids.

**Stereotactic surgery**

The left hemisphere was injected with a lentiviral vector expressing sh-Nurr1 or Nurr1, and the right hemisphere was injected with the scrambled vector as a control. The cannula was withdrawn from the target coordinates 10 min after injection for prevention of regurgitation. After surgery, the incised skin was sealed with Autoclip™ Surgical Suture (Leica Biosystems, Wetzlar, Germany) and animals were monitored on a warm pad until recovery.

**Cell culture**

SH-SY5Y-C99 and SH-SY5Y cells were cultured in Dulbecco’s modified Eagle medium (DMEM) supplemented with 10% fetal bovine serum (FBS) and 0.1 mg/mL penicillin and streptomycin (P/S; Sigma-Aldrich, St. Louis, MO, USA). They were incubated at 37℃ with 5% CO2 in a humidified incubator.

**Western blotting analysis in cultured cells**

SH-SY5Y cells treated with vehicle or AQ were homogenized in RIPA buffer containing protease inhibitor cocktail and sonicated for 5 seconds. After sonication, the samples were centrifuged at 13,000 rpm for 15 min at 4℃. Supernatants were collected as total cell extracts. Equal amounts of protein samples were separated on 8% SDS-PAGE followed by transfer to polyvinylidene difluoride membrane. Membranes were incubated with antibodies against Insulin degrading enzyme (IDE; Abcam, Cambridge, UK) and β-actin (Sigma Aldrich, St. Louis, MO, USA). Immunoreactivity was determined by enhanced chemiluminescence (Amersham Pharmacia Biotech, Buckinghamshire, UK). The image analyzer LAS-3000 (Fuji, Tokyo, Japan) was used for visualization of protein band, and Fujifilm Multi Gauge 3.0 Software was used for quantification of protein band intensities.

***In vitro* assays**

Following treatment with AQ (5, 10 and 15 µM) for 24 h, SH-SY5Y-C99 (S99) cells containing a reporter system for γ-secretase activity were lysed with a passive lysis buffer (Promega, Madison, WI, USA), as previously described (Jin *et al*, 2007). After centrifugation at 13,000 rpm for 15 min at 4℃, the supernatant was collected, and the luciferase activities were measured by a dual-luciferase reporter assay system (Promega, Madison, WI, USA). In addition, *in vitro* peptide cleavage assay was performed for the determination of γ-secretases activity as previously described (Hong *et al*, 2010).

**Immunoperoxidase staining**

Immunoperoxidase labeling was performed as previously described (Jeon *et al*, 2018). Briefly, brain sections were treated with 1% hydrogen peroxide for 15 minutes, and incubated with goat anti-doublecortin (DCX) antibody (1:1,000, Santa Cruz Biotechnology, Dallas, TX, USA), rat anti-Macrophage-1 antigen (Mac-1; 1:1,000, Chemicon, Temecula, CA, USA) and mouse anti-NeuN (1:200, Merck Millipore, Billerica, MA, USA) antibody overnight at 4℃. After 3 PBS washes the tissues were incubated with biotinylated horse anti-goat IgG (1:200, Vector Laboratories, Burlingame, CA, USA), biotinylated goat-anti rat IgG (1:200, Vector Laboratories, Burlingame, CA, USA) and biotinylated horse anti-mouse IgG (1:200, Vector Laboratories, Burlingame, CA, USA). Finally, the tissues were incubated with avidin-biotin-peroxidase complex solution (Vector Laboratories, Burlingame, CA, USA) and visualized using 3,3′-diaminobenzidine solution (Sigma-Aldrich, St. Louis, MO, USA) as a chromogen. An Image-Pro Version 6.0 (Media Cybernetics, Inc., Rockville, MD, USA) was used for quantification of the stained tissues, which were analyzed using both hemispheres of eight brain sections per animal.


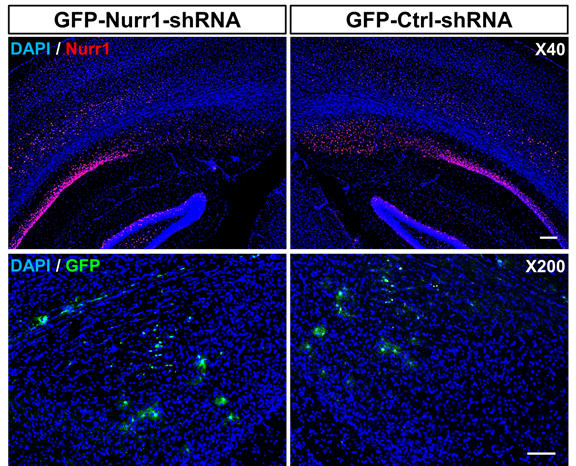


Supporting Information Figure 1 Efficacy evaluation of GFP tagged Nurr1-shRNA in the subiculum. B6SJL mice were injected with lentivirus carrying either GFP-Nurr1-shRNA or GFP-Ctrl-shRNA. After 2 months of infection, there were downregulation of Nurr1 in GFP-Nurr1-shRNA-injected subiculum (upper panel) and expression of GFP in both subiculum (lower panel). Scale bar = 200 μm.


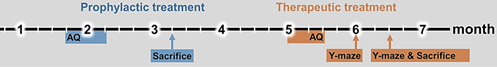


Supporting Information Figure 2 Treatment of 5XFAD mice with the synthetic Nurr1 agonist amodiaquine.

Supporting Information Figure 3. List of human brain samples used for the immunohistochemistry of Nurr1 in hippocampal formation and the western blotting of Nurr1 in hippocampal formation, superior frontal cortex, and substantia nigra. PMI: postmortem interval


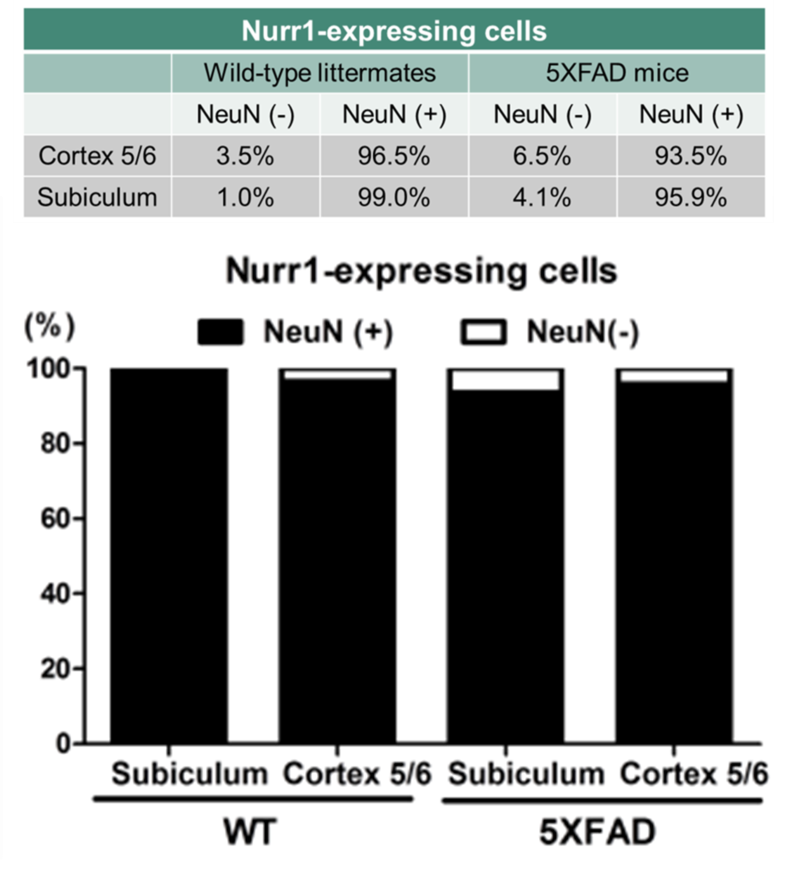


Supporting Information Figure 4. Quantification of Nurr1 (+) NeuN (+) and Nurr1 (+) NeuN (-) cells in the brains of 4 months-old WT littermates and 5XFAD mice. n=862 and 829 for the subiculum and the cortex, respectively, for WT mice and n=480 and 640 for the subiculum and the cortex, respectively, for 5XFAD mice.


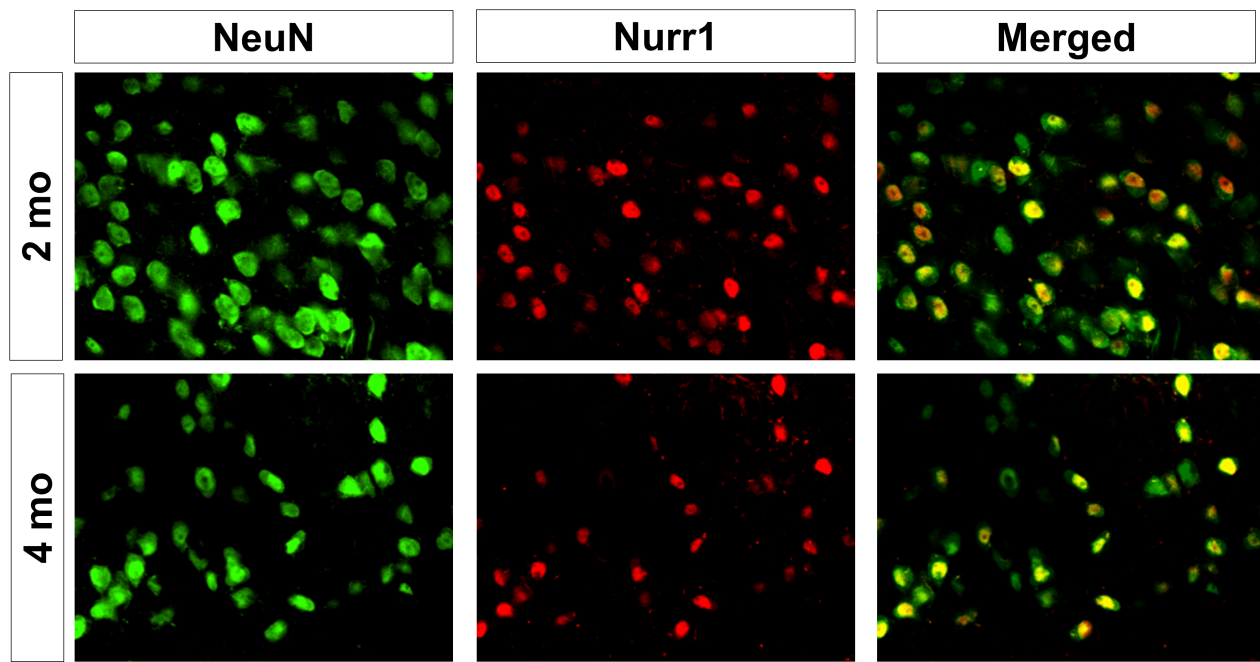


Supporting Information Figure 5. Nurr1 and NeuN double-labeling in the subiculum of WT littermates and 5XFAD mice at 2 and 4 months of ages. Scale bar = 50 μm.

**
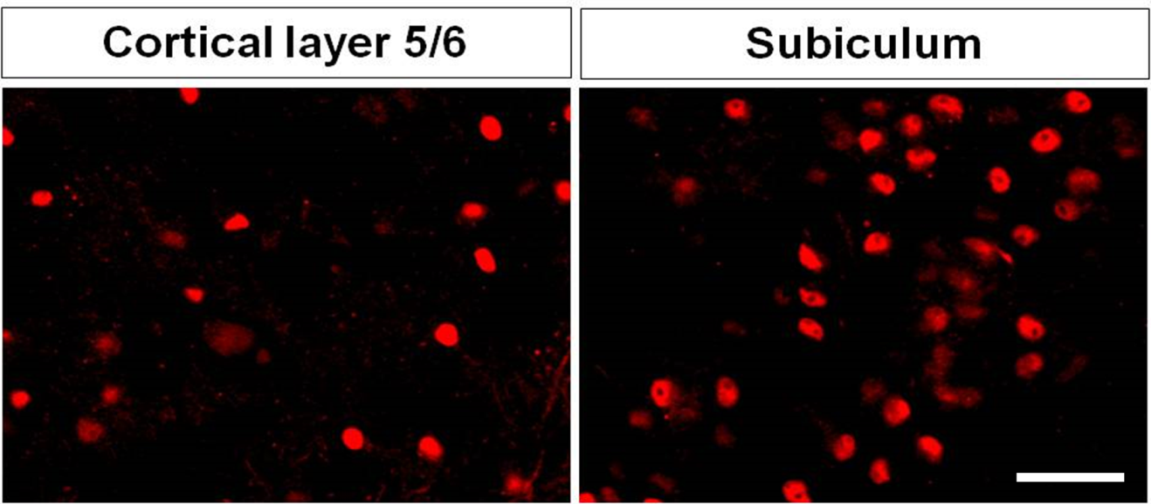
**

Supporting Information Figure 6. Nurr1 expression in deep cortical layers and in the subiculum.


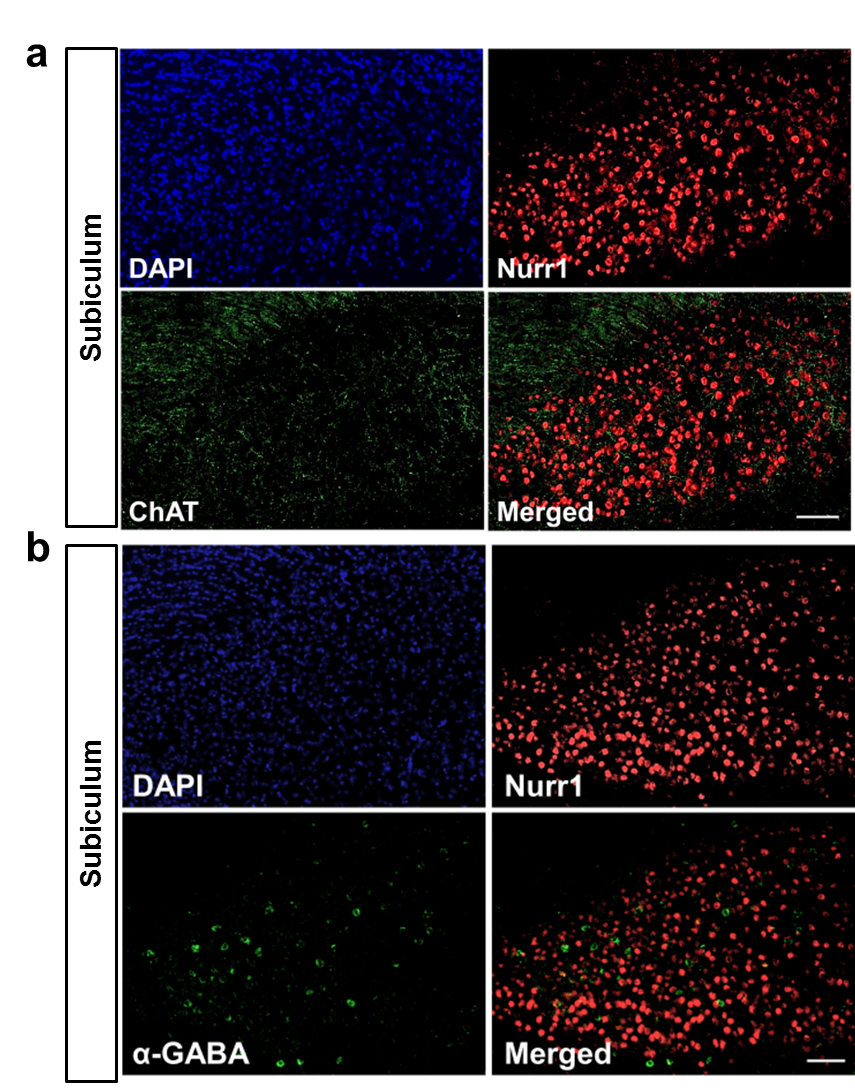


Supporting Information Figure 7. (a) Nurr1 and ChAT (cholinergic cells marker) double-labeling in the subiculum of C57BL/6 mice. (b) Nurr1 and α-GABA (GABAergic cells marker) double-labeling in the subiculum of C57BL/6 mice. Scale bar = 100 μm.


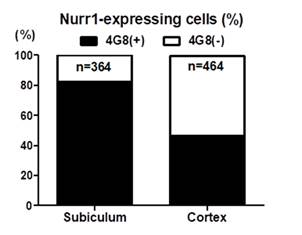


Supporting Information Figure 8. Classification of Nurr1-expressing cells according to Aβ expression in the brain of 2-month-old 5XFAD mice. For quantification, 364 cells in the subiculum and 463 cells in the cerebral cortex from four 5XFAD mice (2-month-old) were counted and analyzed. 82.7% and 46.5% of Nurr1-expressing cells co-expressed Aβ in the subiculum and in the cerebral cortex, respectively.


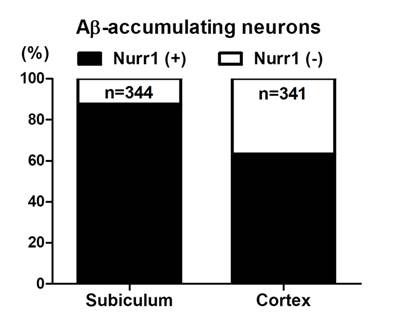


Supporting Information Figure 9. Classification of Aβ-accumulating neurons co-expressing Nurr1 or not. We also quantified the percentage of Aβ-positive cells which co-express Nurr1, by co-staining analyses of 344 cells in the subiculum and 341 cells in the cerebral cortex). 87.5% and 63.3% of Aβ-positive cells co-expressed Nurr1 in the subiculum and in the cerebral cortex, respectively.

**
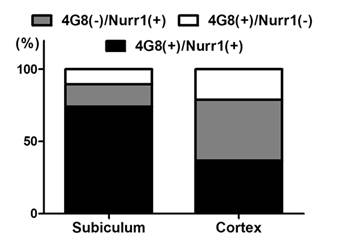
**

Supporting Information Figure 10. Classification of Nurr1 and 4G8-positive cells in the subiculum and frontal cortex in 2 months-old 5XFAD mice. As the expression of APP and PS1 is under the control of the neuron-specific murine Thy-1-promoter in 5XFAD mice (Oakley *et al*, 2006), it is likely that 4G8-positive cells are neurons, not glial cells, and that Aβ accumulation initially occurred in neuronal cells of 5XFAD mice (Moon *et al*, 2012). Taken together, cells in the subiculum and the cerebral cortex of 2 month-old 5XFAD mice can be classified based on the co-expression patterns of Aβ and Nurr1: (1) Aβ-positive Nurr1-expressing cells (subiculum: 74%; cortex: 36.7%); (2) Aβ-negative Nurr1-expressing cells (subiculum: 15.5%; cortex: 42.1%); (3) Aβ-positive Nurr1-negative cells (subiculum: 10.6%; cortex: 21.2%).


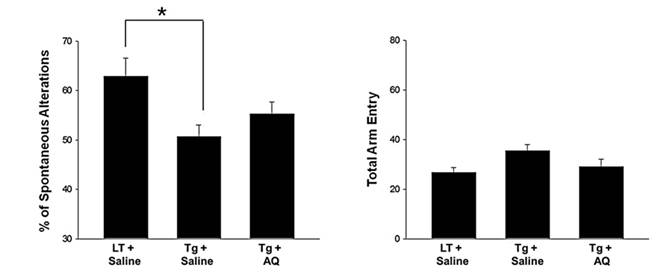


Supporting Information Figure 11. Nurr1 agonist AQ injection regimen in 5XFAD mice. 20 weeks-old 5XFAD mice were administered AQ for 2 weeks (20 mg/kg) and sacrificed at 26 weeks (indicated by red arrow). For AQ therapeutic treatment, we used 5 months-old 5XFAD mice and sacrificed them 4 weeks after the last AQ injection. While AQ treatment failed to significantly improve cognitive function 2 weeks after the last injection (n=10).


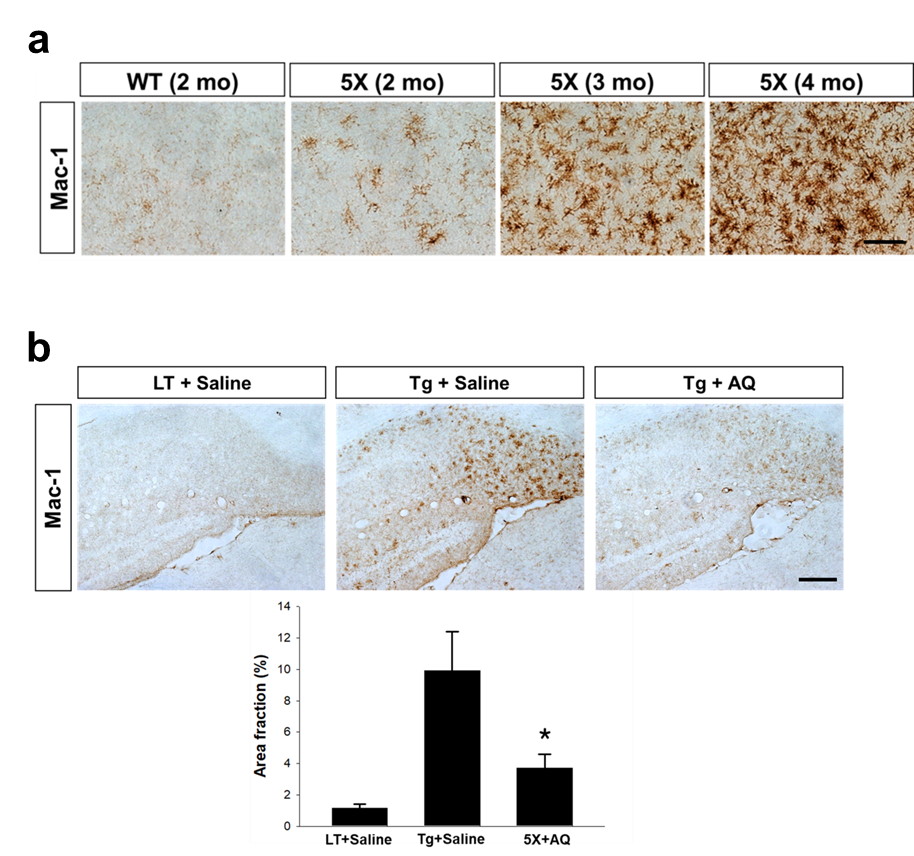


Supporting Information Figure 12. (A) Mac-1 immunoreactivity in the subiculum of 5XFAD mice. Scale bar = 50 μm. (B) Mac-1 immunoreactivity in the hippocampal formation of vehicle or AQ-treated 5XFAD mice and WT littermates. Scale bar = 200 μm.

**
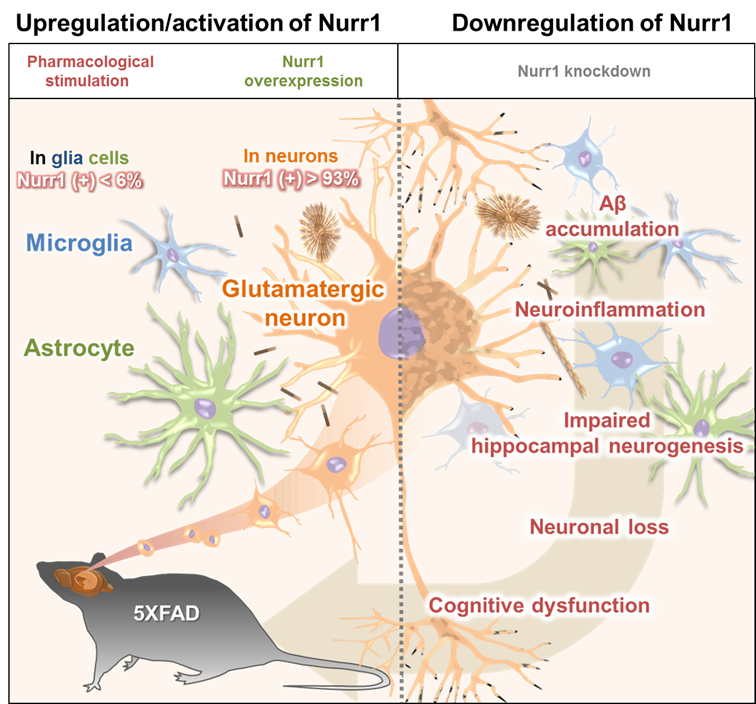
**

Supporting Information Figure 13. Graphical abstract for the crucial role of nuclear receptor Nurr1 in pathogenesis of AD in 5XFAD mice. APP is converted to Aβ by sequential enzymatic cleavage by β-secretase and γ-secretase. The resulting Aβ takes the forms of oligomers, fibers and plaques through self-aggregation and exhibits neurotoxicity. In this series of processes, we demonstrated that the pharmacological activation of Nurr1 by AQ inhibits γ-secretase and increases the expression of IDE. Therefore, the enhancement of Nurr1 through overexpression of Nurr1 and pharmacological stimulation alleviated Aβ accumulation, neuronal inflammation, neuronal loss, impaired adult hippocampal neurogenesis and cognitive impairment. In contrast, knockdown of Nurr1 expression increased the burden of Aβ, neuroinflammation, and neuronal loss. In addition, the expression of Nurr1 was significantly reduced in the brain of AD patients. Remarkably, over 90% of these Nurrl-expressing cells were neurons that mostly expressed glutamate transporter, EAAC1.

References

Hong HS, Hwang JY, Son SM, Kim YH, Moon M, Inhee MJ (2010). FK506 reduces amyloid plaque burden and induces MMP-9 in AbetaPP/PS1 double transgenic mice. *J Alzheimers Dis* **22**(1): 97-105.

Jeon SG, Kang M, Kim YS, Kim DH, Nam DW, Song EJ*, et al* (2018). Intrahippocampal injection of a lentiviral vector expressing neurogranin enhances cognitive function in 5XFAD mice. *Exp Mol Med* **50**(3): e461.

Jin SM, Cho HJ, Jung MW, Mook-Jung I (2007). DNA damage-inducing agent-elicited gamma-secretase activity is dependent on Bax/Bcl-2 pathway but not on caspase cascades. *Cell Death Differ* **14**(1): 189-192.

Moon M, Hong HS, Nam DW, Baik SH, Song H, Kook SY*, et al* (2012). Intracellular amyloid-beta accumulation in calcium-binding protein-deficient neurons leads to amyloid-beta plaque formation in animal model of Alzheimer's disease. *J Alzheimers Dis* **29**(3): 615-628.

Oakley H, Cole SL, Logan S, Maus E, Shao P, Craft J*, et al* (2006). Intraneuronal beta-amyloid aggregates, neurodegeneration, and neuron loss in transgenic mice with five familial Alzheimer's disease mutations: potential factors in amyloid plaque formation. *J Neurosci* **26**(40): 10129-10140.

Pantazopoulos H, Boyer-Boiteau A, Holbrook EH, Jang W, Hahn CG, Arnold SE*, et al* (2013). Proteoglycan abnormalities in olfactory epithelium tissue from subjects diagnosed with schizophrenia. *Schizophr Res* **150**(2-3): 366-372.
